# Multimodal Atlas of Caudate Head Reveals Impact of pTau Burden on Resident Glial Cells

**DOI:** 10.1002/alz70861_109003

**Published:** 2025-12-23

**Authors:** Omar Kana, Kyle J Travaglini, Nadia Postupna, Mariano I Gabitto, Brian Long, Andreas Tjaernberg, Joseph T Mahoney, Ming Xiao, Tejas S Bajwa, Emily Gelfand, Eitan S Kaplan, Jeanelle Ariza, Kimberly A Smith, Delissa McMillen, Anish Bhaswanth Chakka, Jeff Goldy, Erica J Melief, Augustin Ruiz, Stuard Barta, Alana Oyama, Christina Alice Pom, Angela Ayala, Madeline Bixby, Alvin Huang, Naomi Martin, Nasmil J. Valera Cuevas, Paul A Olsen, Josh Nagra, Rushil Chakrabarty, Michael Tieu, Thanh Cardenas, Amy Torkelson, Darren Bertagnolli, Junitta Guzman, Rebecca Ferrer, Jennie L Close, Rebecca D Hodge, Jeremy A Miller, Michael J Hawrylycz, C. Dirk Keene, Ed S Lein

**Affiliations:** ^1^ Allen Institute for Brain Science, Seattle, WA USA; ^2^ University of Washington, Seattle, WA USA

## Abstract

**Background:**

Dysfunction of the Basal Ganglia is implicated in several neurodegenerative diseases such as Parkinson’s and Huntington’s. A substructure of the Basal Ganglia, the caudate nucleus, is observed to have diffuse amyloid plaques in Alzheimer’s disease (AD), in Thal phase III. Additionally, literature suggests the presence of AD ‐related tangles. Functionally, the caudate is known to be involved in cognitive functions impacted by AD such as memory. The caudate also receives signals and has efferent projections to significantly affected regions in AD such as cortex and hippocampus respectively. Despite these connections, caudate nucleus remains understudied in AD.

**Method:**

AT8 (pTau) and 6e10 (Aβ) immunohistochemical staining was performed on the caudate from 42 donors with only canonical proteionopathies and no comorbities. Single nucleus RNA and ATAC‐seq (multiome or singleome) was collected for all donors in the cohort. Spatial transcriptomics was performed on a subset of 5 Thal I‐III and 5 Thal IV‐V donors, with post‐hoc immunostaining of AT8 and 6e10. Cells were labeled using deep learning with a reference caudate dataset from healthy BRAIN Initiative donors. Changes in expression and cell type abundance were modeled in terms of levels of AT8 and 6e10 using Bayesian and general linear mixed effects models respectively.

**Result:**

We identified caudate specific pTau associated abundance increases in astrocyte and microglia types. These microglia types were not the stereotypical disease associated types described in cortex. We also identified pTau associated abundance decreases in oliogodendrocyte subtypes consistent with cortex. Almost all neuronal populations in the caudate show little change in their cellular abundances. Most effects in cellular composition or differential expression were observed specifically with respect to level of pTau and not Aβ.

**Conclusion:**

AD’s impact in caudate head contrasts with established effects on the cortex. Regionally unique increases in certain non‐neuronal populations suggest a caudate specific response to AD. Additionally, little neuronal loss, even with respect to significant pTau pathology suggests either environmental or cellular factors that confer resilience, or distinct pTau conditions in the caudate. Finally, our data suggests that the predominantly diffuse plaques in caudate are not sufficient for a plaque induced response in microglia.

